# Taming Disulfide Bonds with Laser Fields. Nonadiabatic Surface-Hopping Simulations in a Ruthenium Complex

**DOI:** 10.1021/acs.jpclett.1c04143

**Published:** 2022-02-17

**Authors:** Moritz Heindl, Leticia González

**Affiliations:** Institute of Theoretical Chemistry, Faculty of Chemistry, University of Vienna, Währingerstrasse 17, 1090 Vienna, Austria

## Abstract

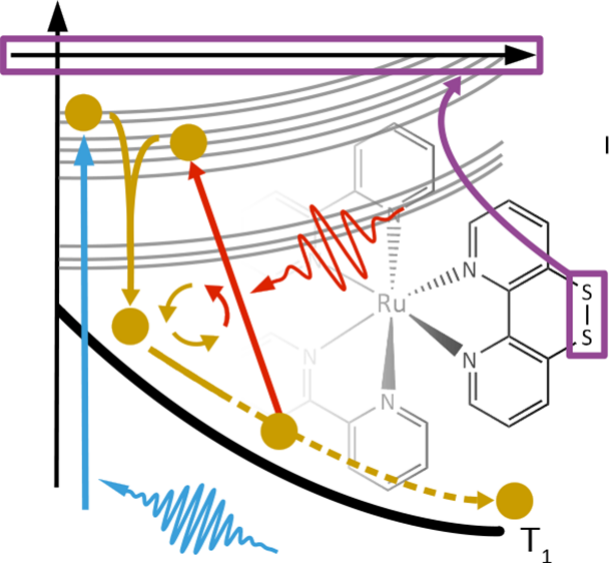

Laser control of chemical reactions is a challenging field of research. In particular, the theoretical description of coupled electronic and nuclear motion in the presence of laser fields is not a trivial task and simulations are mostly restricted to small systems or molecules treated within reduced dimensionality. Here, we demonstrate how the excited state dynamics of [Ru(^S–S^bpy)(bpy)_2_]^2+^ can be controlled using explicit laser fields in the context of fewest-switches surface hopping. In particular, the transient properties along the excited state dynamics leading to population of the T_1_ minimum energy structure are exploited to define simple laser fields capable of slowing and even completely stopping the onset of S–S bond dissociation. The use of a linear vibronic coupling model to parametrize the potential energy surfaces showcases the strength of the surface-hopping methodology to study systems including explicit laser fields using many nuclear degrees of freedom and a large amount of close-lying electronic excited states.

Laser fields can be used to monitor and to modify ultrafast dynamical processes on a molecular level. Experimental progress in laser spectroscopy allows increasing resolution to unravel the flow of excited molecular wave packets.^1^ In parallel, dynamical simulations provide atomistic insight into the potential energy surfaces (PESs) visited by the wave packets.^2^ However, only a few of these simulations include explicit laser fields to excite the system and thus reconcile experimental and theoretical observations.^3−13^

The observed excited state dynamics can be altered using additional pulses tailored to yield outcomes differing from those of the unperturbed dynamics.^14−16^ Here, a classification into weak- and strong-field effects is useful, where weak-field pulses induce transitions between states but do not change the shape of the respective PESs and strong-field pulses influence the dynamics by introducing time-dependent PESs. The large number of successful simulations reported in the literature (see, e.g., refs (17−26)) highlights their versatility. In general, two main avenues can be distinguished to design computationally control pulses.^16^ One is based on exploiting knowledge about the underlying PES and designing laser pulses with a particular reaction pathway. The other uses automated control theories in the form of optimal or local control theory. In optimal control theory, a desired product state or property is maximized over the course of the complete dynamics, relying on a multitude of runs, resulting in an iteratively adapting pulse.^16,19,27^ In contrast, local control theory tries to maximize the chosen state or property in each time step, requiring only a single simulation to yield a locally optimized pulse shape.^17,18,28,29^ The downside of these forms of control is the non-analytical shape of the resulting fine-tuned pulses, often hindering physical interpretation of the excited state mechanisms at work and complicating experimental realization of these pulses.

Previous theoretical work using explicit laser control pulses was mostly done on small systems or by relying on few selected degrees of freedom as well as with limiting participating states, as the sheer number of states and possible relaxation pathways in large systems complicates the excited state dynamics considerably. In this Letter, we use nonadiabatic dynamics simulations in 161 nuclear degrees of freedom to manipulate the excited state dynamics of [Ru(^S–S^bpy)(bpy)_2_]^2+^ (bpy = 2,2′-bipyridine). This transition metal complex is a prototype of experimentally relevant metal-based photosensitizers, with sufficient complexity so that many competing processes take place and control is not straightforward. The peripheral disulfide bridge induces the appearance of metal-to-ligand charge-transfer states localized on the ^S–S^bpy ligand (henceforth labeled MSCT) at energies lower than those pertaining to the standard MLCT states localized on the bpy ligands.^30,31^ However, selective excitation of the MLCT or MSCT states in the ultraviolet (UV) range leads to a decay into the lowest T_1_ state in <200 fs, regardless of the initially populated states^32^ (see Figure 1a). This unselective deactivation is due to the high density of states that form a ladder along which efficient funneling into the T_1_ state occurs. The T_1_ state, with the excited electron located at the ^S–S^bpy ligand, relaxes by weakening the S–S bond and eventually undergoes sulfide extrusion giving a monosulfurated decomposition product.^31^ Such unwanted reaction ruins the promising applications of this complex for excited state proton-coupled multielectron transfer reactivity via the peripheral disulfide/dithiol switch. This work is thus an ambitious attempt to avoid S–S dissociation by altering the natural dynamics of a transition metal complex that possesses a extensive number of nuclear and near-degenerate electronic degrees of freedom.

**Figure 1:**
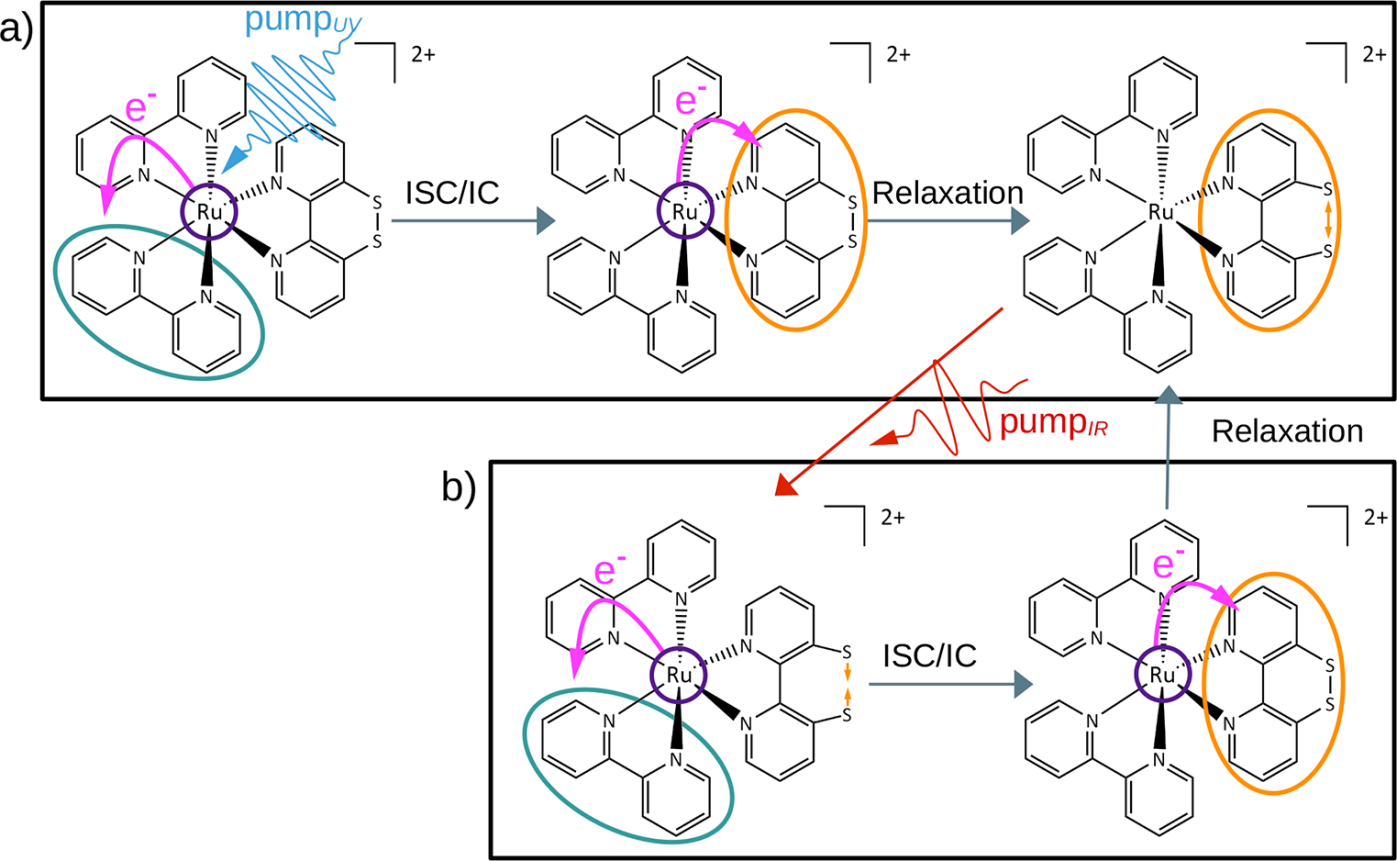
(a) Schematic representation of the natural excited state dynamics of [Ru(^S–S^bpy)(bpy)_2_]^2+^. After irradiation with a UV laser pump pulse at 2.95 eV, mostly MLCT states at the bpy ligands are excited (in petrol green). Within a few hundred femtoseconds, subsequent intersystem crossing (ISC) and internal conversion (IC) lead to the population of states where the excited electron is located at the ^S–S^bpy ligand (orange). From there, the T_1_ state is reached and the S–S bond length increases. (b) Upon addition of a subsequent IR laser control pump field (or a sequence of those), S–S weakening prevented by transferring population from the MSCT back to the MLCT states. This is followed by a number of internal relaxations (IC, ISC, or a combination of both), resulting in localization of the excited electron on the ^S–S^bpy ligand. Upon population of the lowest-energy excited state, an elongation of the S–S bond can be observed.

The simulation and control of the excited state dynamics of [Ru(^S–S^bpy)(bpy)_2_]^2+^ are performed using trajectory surface-hopping dynamics simulations with the SHARC (surface hopping including arbitrary couplings) package.^33−35^ SHARC is an extension of Tully surface hopping,^36^ where nonadiabatic couplings are considered as are spin–orbit couplings and dipole couplings, the latter enabling explicit interaction with laser fields.^4,37^ The trajectories are based on a set of 4000 initial conditions created from a ground state Wigner sampling.^38^ The PESs of the molecule are obtained from a linear vibronic coupling model (LVC),^39,40^ which includes 21/19 singlet/triplet electronic states and 161 vibrational normal modes with parameters described elsewhere.^32^ As an LVC model cannot describe dissociation, we only monitor the initial stretching of the S–S bond. The transition dipole moments that account for the interaction with external laser fields are calculated with the pySOC suite.^41^ Further computational details are available in section S1 of the Supporting Information.

Before undertaking the challenge to modify the excited state dynamics of the complex, we need to investigate the natural response of the molecule using an excitation pump pulse explicitly. To this aim, we employ a pulse with an energy of 2.95 eV, resonant with the main absorption peak of mainly MLCT character,^32^ henceforth labeled as pump_UV_ pulse (Figure S1 and Table S1). We take a Gaussian envelope function with a full width at half-maximum (fwhm) of 50 fs, corresponding to a peak intensity of 75 GW/cm^2^ (1.46 × 10^–3^ au maximum amplitude of the electric field). This pump_UV_ pulse can invert 41% of the population (see Figure 2a) from a set of 4000 trajectories initialized in the electronic singlet ground state. Further details about the laser pulse are provided in section S2. The resulting time-dependent populations are shown in Figure 2a. They are classified according to their electronic character as follows. [Ru(^S–S^bpy)(bpy)_2_]^2+^ is split into four fragments, the central metal atom (M), the two bpy ligands (L and L), and ^S–S^bpy (S). If both the electron and the hole in the excitation are located on the same fragment, the state is a centered state (that is, metal-centered MC, ligand-centered LC, or SC), while if electron and hole are located on different fragments, the states show charge-transfer (CT) character (i.e., MLCT, MSCT, LMCT, SMCT, LSCT, SLCT, and LLCT). This analysis is done from the transition density matrices using TheoDORE.^42^

**Figure 2:**
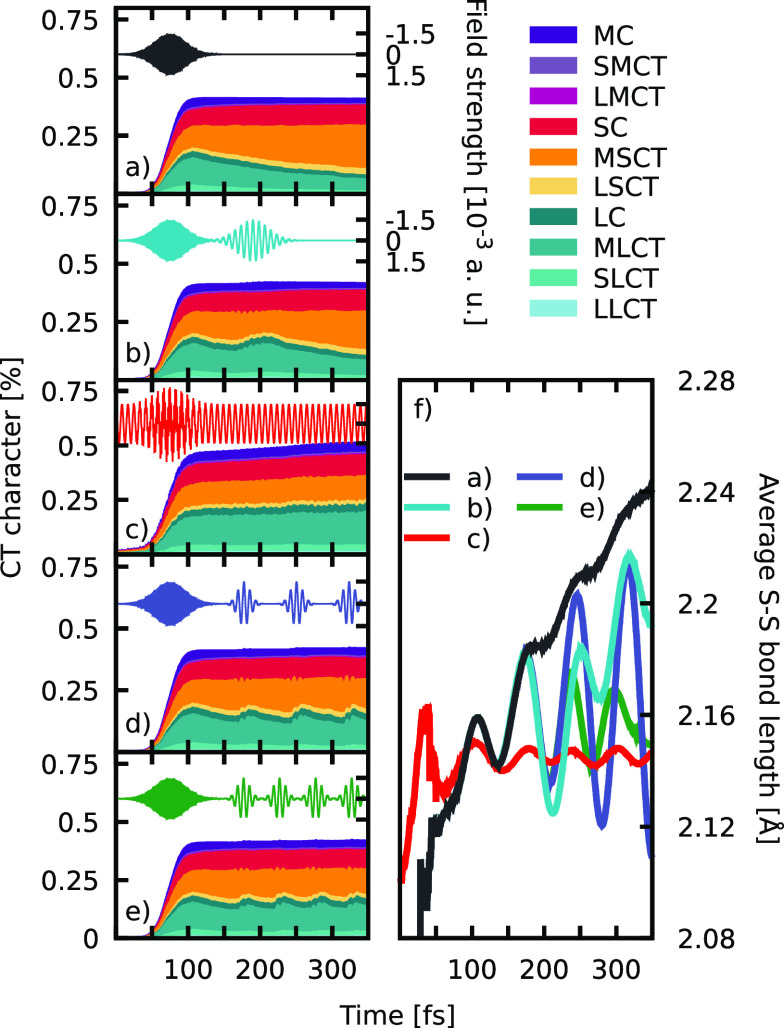
(a–e) Time-dependent populations classified according to their charge-transfer character influenced by the pulse sequences depicted. (f) Time evolution of the average S–S bond length for all excited trajectories simulated using the pulse sequences depicted in panels a–e.

According to their electronic character, the pump_UV_ pulse excites a mixture of MLCT, MSCT, and SC states (Figure 2a). However, after ∼100 fs, there is a clear decline of the MLCT contribution in favor of MSCT states. This decline agrees with the decrease previously found in the simulations of ref (32) that instead of a pump pulse used an instantaneous excitation (i.e., placing all of the trajectories at *t* = 0 fs directly in the electronic excited states, reminiscent of the reflection principle in wave packet dynamics^43^). Both sets of dynamics are yet different at initial times: upon instantaneous excitation,^32^ the initial distribution of CT states is dominated by MLCT states, which then undergo ultrafast transfer into MSCT states; using a finite pulse, there is an almost equal mixture of MSCT+SC and MLCT states at the peak of the pulse. The latter is due to the delayed excitation implicit within the finite pump pulse, where any excited trajectory will already start the ultrafast relaxation toward MSCT states while other trajectories are being excited a bit later, resulting in a mixture of populations at the peak of the pulse.

Population of the T_1_ state is associated with an increase in the equilibrium S–S bond length, which in the employed LVC model goes from 2.11 Å in S_0_ to 2.34 Å in T_1_. Note that the latter value is underestimated with respect to TD-DFT reference calculations,^32^ due to the harmonic character of the LVC potentials. In any case, the elongation of the average S–S bond length for all excited trajectories (Figure 2f) can be taken as a clear indication of S–S dissociation leading to eventual sulfide extrusion.^31^ The observed S–S bond stretching is not linear but goes in steps. The initial peak can be explained by two concurrent effects. On one hand, a coherent increase in the S–S bond length for the excited state trajectories is visible as trajectories are promoted out of the S–S equilibrium bond length in the S_0_ state. On the other hand, the use of a Gaussian-shaped pump pulse imprints a Gaussian shape in the evolution of the properties of the excited wave packet (section S3). Indeed, the first maximum of the S–S bond length average located at 104.5 fs has exactly the width of the intensity of the laser pulse (Figure S2) and arises 29.5 fs after the maximum of the pump pulse. Therefore, excited trajectories take on average 30 fs to increase the S–S bond length from the initial length. Subsequent maxima are roughly 75 fs apart, corresponding to the time needed for a single oscillation of the disulfide bond. Those subsequent maxima undergo a visible broadening at later times due to an increase in the population of the T_1_ state and relaxation to the respective minimum structure featuring the weakened S–S bond. The weakened disulfide bond in turn increases the time needed for a single oscillation, resulting in a broadening in time for reaching the S–S bond length maxima as more and more trajectories fall out of sync.

Looking at the PESs that connect S_0_ with the T_1_ minimum structures (Figure 3), one can see that all states but T_1_ are destabilized when approaching the T_1_ minimum. Thus, once the T_1_ state is populated, relaxation is barrierless and almost ballistic. The high density of states surrounding the initially excited states opens up a multitude of different branching pathways, all eventually leading to the T_1_ state, where the excited electron is located at the ^S–S^bpy ligand. To modify this ultrafast decay toward states that involve the ^S–S^bpy ligand and ultimately lead to an S–S bond length elongation, a suitable target needs to be defined. Keeping in mind that the T_1_ state is of MSCT+SC character and knowing that MLCT states are found at energies higher than their MSCT counterparts, we set our control goal to increase the population of states located at the plain bpy ligands through the dynamics. Due to the large number of states, different onset times, and possible relaxation routes, no suitable laser pulse can be derived from a mere inspection of the PESs in Figure 3. Instead, and borrowing from local control theory,^44^ we define the time-dependent target quantity *p*_L_(*E*_*βα*_, *t*)
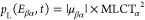
1where β is the electronic state the trajectory currently populates (active state) that will change over the course of the dynamics for each trajectory and α is another excited state with a particular percentage of MLCT character (MLCT_α_). The transition dipole element between α and β (μ_*βα*_) is proportional to the ease of a transition between both states, and *E*_*βα*_ is the energy difference between the active state and state α indicating if an excited state is located above the currently active electronic state (positive value of *E*_*βα*_) or below it (negative value of *E*_*βα*_) and therefore reminiscent of the frequency needed for resonance between β and α. This time-dependent measure can be evaluated for each state and each time step for all trajectories to obtain a map that showcases which laser pulses are likely to excite population into the MLCT band.

**Figure 3:**
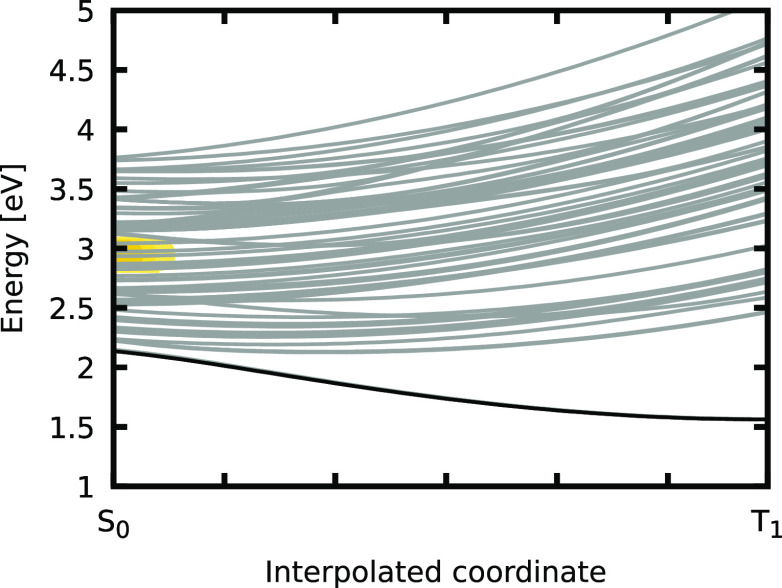
Potential energy plot for all considered states in a spin-mixed representation along the interpolation of internal coordinates from the S_0_ to the T_1_ minimum energy geometry. The lowest-energy excited state is colored black, and the region where the pump pulse will promote strongest is marked in yellow. Note that the electronic ground state is not shown.

To further increase the selectivity between MLCT and MSCT or SC states, we expand the quantity *p*_L_(*E*_*βα*_, *t*) to *p*(*E*_*βα*_, *t*), defined as
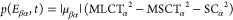
2where MSCT_α_ and SC_α_ are the contributions of MSCT and SC character, respectively, of excited state α. We collected *p*(*E*_*βα*_, *t*) values for 1000 trajectories propagated during 250 fs, starting in S_0_, and using the pump pulse described above. The resulting *p*(*E*_*βα*_, *t*) values have been convoluted in energy with Gaussian functions of 0.3 eV fwhm, yielding Figure 4.

**Figure 4:**
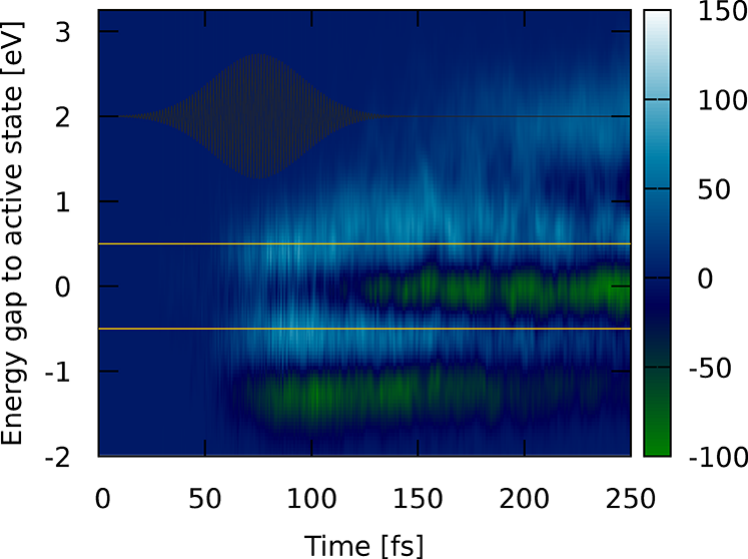
Values of *p*(*E*_*βα*_, *t*) initiated by the pump_UV_ pulse, reminiscent of the density of trajectories that could transiently be excited from the current active state to either MLCT states [positive *p*(*E*_*βα*_, *t*) values colored from light blue to white] or MSCT+SC states [negative *p*(*E*_*βα*_, *t*) values colored green]. The *y*-axis is the energy needed for a resonant transition with the active state. The pump pulse is colored black, and a viable laser frequency for exciting MLCT states is indicated with horizontal gold lines.

We see that around *t* = 75 fs, MLCT bands (light blue) that could be transiently excited build around 0.5 eV above and below the currently active state (see gold lines). Note that the value of 0.5 eV corresponds to excitation into excited states located 0.5 eV above the state in which the trajectory is currently evolving, while a value of −0.5 eV stands for spontaneous emission into states 0.5 eV below the active state. MSCT+SC states (green) instead are found at lower energy differences (1.2 eV below the active states), corroborating their overall stabilization with respect to the MLCT states. Throughout the dynamics, the energetic position of these MLCT and MSCT+SC bands stays constant, fading out slowly only toward the end of the dynamics as they represent lower-energy states that can be accessed from rather high energy states; these higher-energy states become less and less populated the longer the dynamics continues due to nonadiabatic relaxation to lower-energy states. An additional MSCT+SC band appears at the same energy as the active states (0 eV in Figure 4), representing the fact that a large number of trajectories are now found in the MSCT+SC band of states from where other MSCT+SC states can be excited with <0.2 eV. Finally, we can see that a MLCT band arises around 200 fs at ∼2 eV. This additional band represents trajectories that are very close to the T_1_ minimum energy geometry from which the MLCT band is far in energy (∼2 eV).

The analysis of the measure *p*(*E*_*βα*_, *t*) reveals whether the active trajectory can be excited to either MSCT+SC or MLCT states at a given time and which wavelengths would be best suited to do so. Using this information, two different control pulses capable of promoting the active trajectory to an MLCT state can be envisioned. In one, a pulse with a central frequency of 2 eV could be used to excite trajectories close to the T_1_ minimum geometry back to MLCT states, from where the S–S bond would contract. Figure 4 shows that this pulse might be a viable option toward the later stages of the dynamics where this light blue band becomes visible at 2 eV as more trajectories start to populate the T_1_ minimum structure. A second realization for a control pulse could act at a much lower frequency (0.5 eV) and target trajectories in the process of descending down the ladder of states toward the low-lying MSCT+SC states. The respective energy gap (±0.5 eV) is marked in gold in Figure 4, where one can see that this pulse would consistently promote trajectories to MLCT states throughout the dynamics. However, when appraising the benefits and drawbacks of the two possible control pulses, one must realize that a 2 eV pulse would also act as an additional pump pulse inverting the S_0_ population, further complicating the ensuing dynamics. Therefore, the 2 eV pulse was discarded and only the infrared (IR) 0.5 eV pulse will be used besides the original pump_UV_ pulse. We note that surface hopping cannot account for vibrationally excited states that could potentially be excited by an IR pulse within the electronic ground and excited states, so these are excluded.

Accordingly, the following two-pulse setup is envisioned: the pump_UV_ pulse used previously to excite the population from S_0_, followed by a 0.5 eV pulse (labeled pump_IR_), with again a fwhm 50 fs Gaussian envelope and a maximum intensity of 75 GW/cm^2^ (corresponding to a 1.46 × 10^–3^ au maximum amplitude of the electric field). The schematic influence of the expected dynamics when applying the pump_IR_ to control the dynamics of [Ru(^S–S^bpy)(bpy)_2_]^2+^ is shown in Figure 1b. The delay time (τ) between UV and IR pulses is defined as
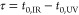
3where *t*_0,IR_ and *t*_0,UV_ are the centers of the IR and UV pulses, respectively. This delay is set to 115 fs (section S3 and Figure S3) to guarantee a distinct separation between the initial UV pump and the IR pulse. We note that because the transition dipole moments of [Ru(^S–S^bpy)(bpy)_2_]^2+^ are very large and they are paired with an intense laser field, the resulting dynamics will be a combination of weak- and strong-field effects. The evolution of the states triggered by the pump_UV_–pump_IR_ pulse sequence is shown in Figure 2b. During the duration of the pump_IR_ pulse, an increase in the level of MLCT character is visible, accompanied by a decrease in the level of MSCT character, resulting in a distribution of MLCT to MSCT+SC characters similar to the distribution at the end of the pump_UV_ pulse. Accordingly, the average S–S bond length of excited trajectories (Figure 2f) undergoes a large decrease during the time the pump_IR_ pulse is on. Both observations indicate a successful population of MLCT states during the pump_IR_ pulse, which in turn leads to a hardening of the S–S bond. However, as the pump_IR_ pulse ends, repopulation of the MSCT states is observed paired with an increase in the average S–S bond length.

As a next attempt to stop the decay toward the T_1_ state completely, the pump_IR_ pulse was substituted with a continuous wave with a frequency of 0.5 eV providing a permanent drain from MSCT to MLCT. This continuous wave overlaps with the pump_UV_ pulse, so that it excites few trajectories at earlier times than the pulse(s) of panels a and b of Figure 2, as one can see in Figure 2c. After the pump_UV_ pulse ends, more and more trajectories are excited with 51% population inversion at 350 fs. As intended, the decay of the MLCT character is completely blocked and the ratio of CT states remains constant after the maximum of the initial pump_UV_ pulse. Furthermore, the S–S bond length (Figure 2f) oscillates around a value of 2.145 Å, only slightly longer than the equilibrium value (2.11 Å). Thus, this permanent IR continuous wave traps the excited population effectively in a mixture of states where the electron is located at any of the three ligands without relaxing to the T_1_ minimum structure.

Finally, we investigate whether the continuous wave can be efficiently replaced by a train of short pump_IR_ pulses, allowing for a more targeted modulation of the excited state dynamics, where each single IR pulse would promote population of MLCT states accompanied by a reduction of the S–S bond. To position the pump pulses, we use the oscillations of the S–S bond length, so that the pump pulses are placed at the respective maxima. We use three pump_IR_ pulses with a fwhm of 20 fs, separated from each other by 73 fs with the first acting with a τ of 102 fs between the pump_UV_ pulse and the first IR pulse. The resulting dynamics are shown in Figure 2d. As one can see, the level of MLCT character increases during the duration of all three laser pulses at the expense of the MSCT contribution and the S–S bond decreases strongly for each pulse hitting the complex. However, concomitant with each decrease in S–S bond length, there is a stronger increase, leading to even stronger coherent oscillations after each pulse. This unwanted behavior can be rationalized by two accumulating effects. One relates to the timing of the pump_IR_ pulses, which acting at the maximum of the S–S bond length for most trajectories, promotes the system to populate MLCT states, but the accompanying hardening of the bond results in an increase in kinetic energy along the S–S bond vibration, resembling a swing on a playground where an extra force is applied when the maximum height is reached. The second is that the strong pump_IR_ pulses can increase the level of synchronization between more and more trajectories as the PESs shift with the time-dependent slow oscillations of the pulse. As a result, this train of IR pulses is not useful for preventing S–S dissociation.

An alternative is to set the center of the IR pulses asynchronous to the S–S bond oscillations to obtain the opposite of the swing effect described above. To this aim, another train of pump_IR_ pulses is simulated, starting from the same first IR pulse with all subsequent laser pulses acting with a τ of only 50 fs. This way, the first pump_IR_ pulse initiates the swing, which is then damped by the next pulse acting before a full oscillation of the S–S bond can be completed, effectively removing kinetic energy from the vibration. The time-resolved population is depicted in Figure 2e. A larger proportion of MLCT states and a decrease in the average S–S bond length are achieved, when compared to those of the previous pulse sequence. The asynchronous timing of the pulses with respect to the S–S bond oscillations results in an effective shortening of the average S–S bond length compared to the uncontrolled dynamics, giving an average bond length that oscillates around a value of 2.16 Å.

At this point, it is appropriate to discuss the limitations of the model employed. We were able to perform laser-induced nonadiabatic simulations of a system featuring 161 nuclear degrees of freedom within a total of 78 electronic states relying on a series of approximations. (i) The underlying LVC model is valid only close to the ground state equilibrium geometry and in rigid molecules, such as transition metal complexes. However, adjustments to the LVC model had to be made to allow the correct energetic and structural position of the T_1_ state, as detailed in ref (32). Within these constraints, the model is expected to give a reasonable dynamical behavior during the time of the simulation. (ii) One serious limitation is that due to the use of harmonic potentials, LVC models cannot describe bond dissociation.^45^ Thus, in this work our analysis focuses on only the early elongation of the S–S bond. We investigate how the molecule approaches the T_1_ minimum structure optimized by TD-DFT, which is the first step toward dissociation. As a consequence, we cannot predict the time scale or quantum yield of actual dissociation, as none of our simulated S–S bond oscillations can dissociate. Instead, our laser control strategy is targeted to counteract the relaxation of all trajectories on the way to the T_1_ minimum. (iii) Spin–orbit couplings and the complete dipole matrix are taken only from the reference geometry, and the respective matrices at other geometries are formed via mixing of states. This approximation is valid for slowly changing properties, as it is the case of spin–orbit couplings. For the dipole moments, it is not possible to quantify the error introduced, both within the pump_UV_ and within the pump_IR_, which acts on the molecule farther from the Franck–Condon position. However, we assume that the results should not change qualitatively, the major difference being the amount of trajectories that would be excited to the target states, a problem that can be solved by adjusting the laser intensity. (iv) Furthermore, surface hopping has its own inherent limitations due to the use of independent classical trajectories. The independent simulation of each trajectory leads to a complete neglect of nuclear interference terms that might be especially strong in the presence of laser fields, inducing a coherent set of excited state pathways. The classical nature of each trajectory means that the interaction with the pump_IR_ pulse misses the potential excitation of vibrational states. Previous investigations using surface hopping in the presence of a laser indicate that caution should be exercised in the choice of parameters^11,13,46^ or when using long laser pulses.^5^ Due to the different lengths of the employed laser pulses, different deviations from exact results could occur, with the largest deviation predicted for the continuous wave.

In conclusion, in this Letter we show how the excited state dynamics of [Ru(^S–S^bpy)(bpy)_2_]^2+^, a derivative of the well-known photosensitizer [Ru(bpy)_3_]^2+^, can be modified using laser pulses in the context of surface-hopping trajectory simulations. We show that a simple IR pulse can transiently excite the population away from the pathway to the T_1_ minimum energy structure, which is characterized by a weakening of the S–S bond that finally leads to dissociation. However, after the pulse ends, the natural course of the dynamics turns on, steering back toward the T_1_ minimum and thus leading to S–S bond stretching. By replacing the infrared pulse by a continuous wave of the same frequency, we demonstrate that the distribution of populated states and the S–S bond length can be frozen almost at the ground state equilibrium value preventing dissociation. Similar effects can be achieved by using a train of pulses, where depending on the interval between two pulses, the oscillations of the S–S bond can be better synchronized or kept at a strongly reduced level close to the modifications achieved using a continuous wave. By ultimately avoiding sulfide extrusion, such a compound could be subsequently reduced by exploiting the two-electron, two-proton nature of the disulfide/dithiol interconversion and find applications in solar fuel generation.

This example suggests that control of excited state dynamics in large molecular systems can be successfully modeled in very high dimensionality and can hopefully inspire laser control experiments. The presented approach could be extended to other transition metal complexes, where manipulating the different electronic states can be useful for breaking or holding a particular bond or pushing electron transfer in a particular direction.

## Supporting Information Available

Further computational details, definition of the laser fields used, additional simulations and analysis of the S–S bond length through the dynamics, and time delays among the pulses (PDF)
