# Supplementary material for: Taming Disulfide Bonds with Laser Fields. Nonadiabatic Surface-Hopping Simulations in a Ruthenium Complex
Source: J Phys Chem Lett. 2022 Feb 17;13(8):1894–900. doi: 10.1021/acs.jpclett.1c04143 (PMC8900122; doi:10.1021/acs.jpclett.1c04143)
Supplement: Supplementary file 1 — jz1c04143_si_001.pdf [file jz1c04143_si_001.pdf]

Supporting Information: Taming Disulfide Bonds with Laser Fields. Nonadiabatic Surface-Hopping Simulations in a Ruthenium Complex

Moritz Heindl, Leticia González
Institute of Theoretical Chemistry, Faculty of Chemistry, University of Vienna, Währingerstr. 17, 1090 Vienna, Austria

February 6, 2022

Contents

S1 Further computational details 1

S2 The laser pulses 2

S3 Coherent increase in the S–S bond length 4

S4 Time delay analysis 5

S5 Random laser polarisation 5

S6 Influence of adapting kinetic energy on a hop 7

S1 Further computational details

All the nonadiabatic dynamics simulations have been carried out using the program package SHARC 2.1.[1] The nuclear and electronic propagation was performed in a completely diagonal basis where both the off-diagonal coupling terms of the transition dipole moments with the external field and the spin-orbit couplings are projected onto the diagonal of the Hamiltonian, forming a set of spin-mixed states.[2,3] In the context of laser fields, such a procedure is known as the instantaneous adiabatic representation or instantaneous Born-Oppenheimer representation.[4] Such a basis has been found to be beneficial compared to keeping the laser interactions as off-diagonal elements in the presence of strong external fields[5] but has also been shown to give nonphysical results in other cases where a Floquet basis was found to be superior.[4,6,7] A time step of 0.25 fs has been employed for the nuclear propagation while the electronic wave function was propagated using time steps of 0.01 fs in a locally diabatic basis.[8] The simulations

carried out to find suitable wave lengths for control of the excited state dynamics have been run for 250 fs, while all other simulations were run for 350 fs.

Decoherence of the electronic wave function on different states has been considered with the energy-based criterion acting in every time step using a decoherence parameter of 0.1 Hartree.[9,10] Commonly, the kinetic energy of surface hopping trajectories is adapted at a hopping event to counteract the change in the potential energy experienced at the hop, hence, conserving the total energy of each trajectory.[11,12] In the presence of laser fields, this is complicated by the fact that energy uptake or dissipation in the form of photon absorption or stimulated emission is possible, resulting in changes to the total energy. To differentiate between both types of hops (light-induced and not light-induced), a number of possibilities exist,[2,13] but none of them were found suited to deal with the here investigated transition metal complex in the diagonal representation and the sets of pulses employed. Additionally, modifying the kinetic energy along the non-adiabatic coupling vector –which is considered the best direction to rescale the kinetic energy along[12,14,15]– is complicated in the presence of states of different multiplicities, and this is not remedied by the use of a set of mixed states.[16] Therefore, it has been decided that the kinetic energy at a transition between states is not adapted, even if in the literature such decision showed deviations in comparison with quantum simulations.[12,17,18] In order to estimate how much the current dynamics is affected by this choice, a comparison is shown below in Section S6.

S2 The laser pulses

The interaction of the molecule with laser fields is treated within the semi-classical dipole approximation, where the field is classical and the electrons are quantum mechanical objects. A uniform field within each time step is assumed. The multipole expansion of the interaction of the field with the molecule is truncated to linear terms, i.e. to the interaction with the transition dipole moments of the system. Interactions due to the overall charge of the molecule are neglected as these would be identical for all considered states because the number of electrons does not change within the simulations. Accordingly, the simulated interaction ($\hat{V}_{ext}$) takes the form of

$$\hat{V}_{ext} = -\hat{\mu}(\mathbf{R}) \cdot \epsilon(t) \tag{1}$$

with $\hat{\mu}(\mathbf{R})$ being the dipole matrix and $\epsilon(t)$ the time-dependent field. The field can be written as a sum of N_L laser pulses as follows

$$\epsilon(t) = \sum_{i}^{N_L} \mathbf{p}_i \cdot \epsilon_i^0 \cdot \cos(\omega_i(t - t_{0,i}) + \eta) \exp\left[-4 \cdot \ln 2 \left(\frac{t - t_{0,i}}{FWHM_i}\right)^2\right]. \tag{2}$$

Here $\mathbf{p}_i$ is the polarization vector of laser pulse i with a maximum amplitude of ϵ_i^0 and frequency ω_i. The phase factor η is set to zero. The envelope function is a simple Gaussian function centered at $t_{0,i}$. For all simulations, a linearly polarized field along the x direction was used, as the molecule showed the largest transition dipole moments along this axis (which in a laboratory fixed molecule, it points from the central metal atom towards the disulfide bridge of $^{S-S}$bpy). The estimation of the $p(E_{\beta\alpha}, t)$ values to determine suitable control pulses also include transition dipole moments along x. In order to verify that the observed dynamics is not an artifact of the assumed alignment of the simulated ruthenium complex, the influence of changing the polarization from x direction to a random polarization is discussed in Section S5.

All laser fields used in the main manuscript are depicted in Figure S1 with laser parameters collected in Table S1.

	ϵ_i^0 [GW/cm^2]	ω [eV]	$t_{0,i}$ [fs]	$FWHM_i$ [fs]
a)	75	2.95	75.0	50.0
b)	75	0.50	190.0	50.0
c)	75	0.50	0.00	∞
d)	75	0.50	177.5/250.5/323.5	20.0
e)	75	0.50	177.5/227.5/277.5/327.5	20.0

Table S1: Laser parameters of the lasers employed in panels a-e of Fig S1.

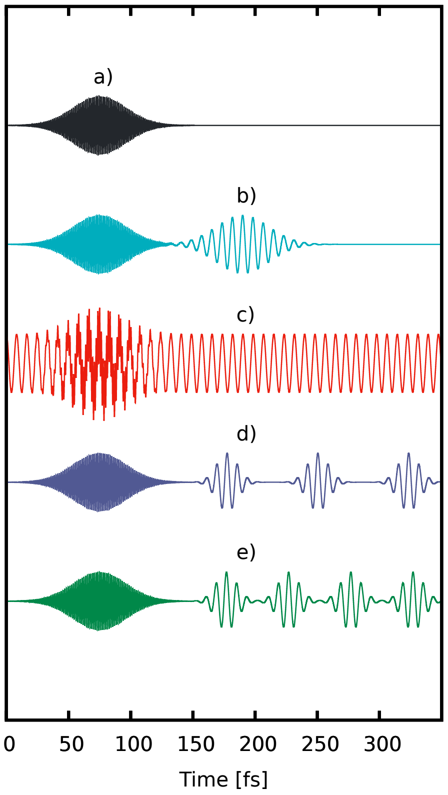

Figure S1: Laser fields used in the main manuscript. Parameters listed in Table S1 .

S3 Coherent increase in the S–S bond length

Excitation of $[Ru(^{S--S}bpy)(bpy)_2]^{2+}$ with the pump$_{UV}$ pulse triggers coherent oscillations in the average of the S–S bond length, see Figure S2. The average S–S bond length increases until reaching a first maximum located 29 fs after the maximum of the pump pulse. Following this first maximum, the average S–S bond length oscillates to lower values before entering a stepwise and recurring increase in average as more and more trajectories decay towards the T_1 minimum energy structure, where the S–S bond drastically weakens.

When investigating this oscillatory behavior, we found that the first oscillation of the average bond length almost exactly coincides with the shape of the applied intensity I ($I \propto \epsilon(t)^2$). As mentioned in the main text, a two-fold mechanism is at work here: I) First, the amount of excited trajectories changes during the length of the pump pulse. Each excited trajectory initiates its dynamics at a different point in time, resulting in different absolute time scales at which the T_1 minimum is reached. The more trajectories get excited in a single time step, the stronger the influence of these coherently excited trajectories on the average S–S bond length, resulting in an intensity shaped oscillation. II) Second, the S–S bond length moves coherently in the initially excited states. The interested reader can go to Figure S5c where no explicit laser pulse is used to excite the trajectories but instead the trajectories are directly projected in the electronic excited states at time zero. In this case, a very similar pattern of rising average S–S bond length is observed, albeit the oscillations are sharper than when using an explicit UV laser pulse.

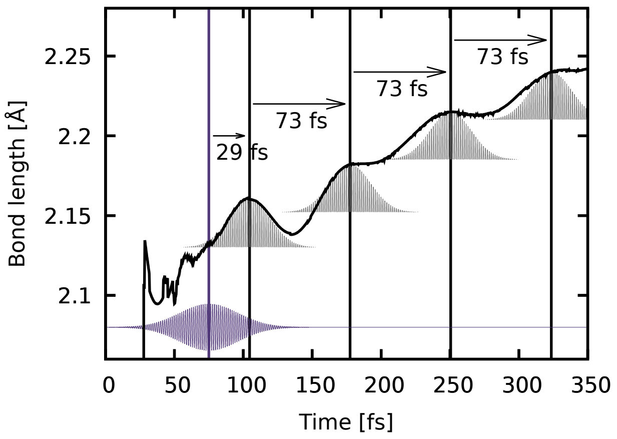

Figure S2: Average S–S bond length (black) after a pump$_{UV}$ excitation. The laser field $\epsilon(t)$ is plotted in purple. The grey shapes fitted into the maxima along the S–S bond average correspond to the (scaled) intensity ($\epsilon(t)^2$) of the pump pulse, which was manually moved to be centered at the respective maxima.

Overall, we can conclude that the oscillations are due to differences in the equilibrium point of the ground and the excited states, while the broadening of the observed oscillation is due to the employed pulse. The oscillation has a recurrence time of ca 73 fs, close to experimentally determined disulfide bridge bond vibrations (between 65-77 fs).[19] At later times, the oscillations flatten out as trajectories follow different relaxation pathways, resulting in slightly shorter or longer bond oscillations, until more trajectories show longer bond oscillations as the S–S bond weakens.

S4 Time delay analysis

To see how sensitive the excited state dynamics is to the position of the broad FWHM=50 fs pump$_{IR}$ pulse, the effect of several time delays (τ) have been investigated using 4000 trajectories. Besides τ of 115 fs, presented in the main manuscript, we have simulated τ=95 and 135 fs. As shown in Figure S3, the S–S bond length is different, with a smaller dip for τ = 95 fs compared to τ = 115 fs. For a τ = 135 fs the dynamics is similar as for τ =115, indicating that with the employed broad pulse featuring a FWHM of 50 fs, the exact position of the IR pulse is not important to see a reduction in the average of the S–S bond length.

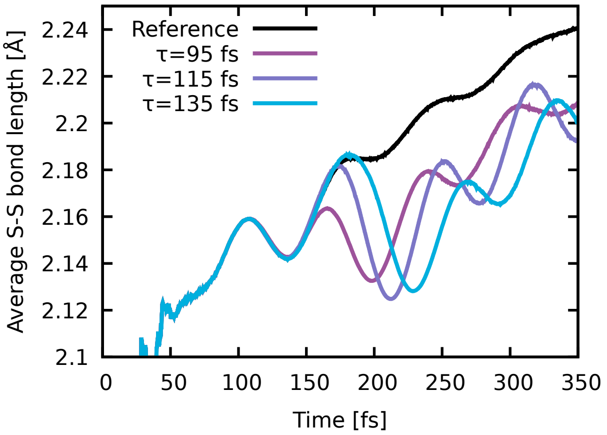

Figure S3: Average S–S bond length for different time delays (τ) between the pump$_{UV}$ and the pump$_{IR}$ pulses (FWHM=50 fs). The reference uses only pump$_{UV}$.

S5 Random laser polarisation

All simulations presented up to now have been conducted using linearly polarized electric fields in the x-direction, because it corresponds to the direction of the largest transition dipole moment, and and thus the initial excitation is maximum along this direction. In order to estimate the effect of the polarization, two computational approaches can be conceived. One is to randomly assign the spatial orientation of each molecule while the electric field acts along x. Another is to keep the orientation of the molecule and randomly adapt the polarization vector of the electric field. We chose the latter approach as it is simpler to implement, especially since each rotated molecule would need to be rotated back into the reference system of normal modes employed in the LVC model.

Using a random polarization for the electric field, two new simulations are set up (Figure S4c and S4d) and compared to those of the main manuscript in Figure 2a and 2b (shown again in Figure S4a and S4b). 4000 trajectories are set up in all cases, each having a completely randomized polarization vector for the complete field (i.e. in a two-pulse setup, both pulses share the same polarization vector) and normalized to still yield a maximum of 75 GW/cm^{-2} intensity. For the case of only pump$_{UV}$, less population is excited with randomly polarized light (26 % compared to 41 %), corroborating that the largest transition dipole moment is located along the x direction. The character of the states populated in both cases (Figure

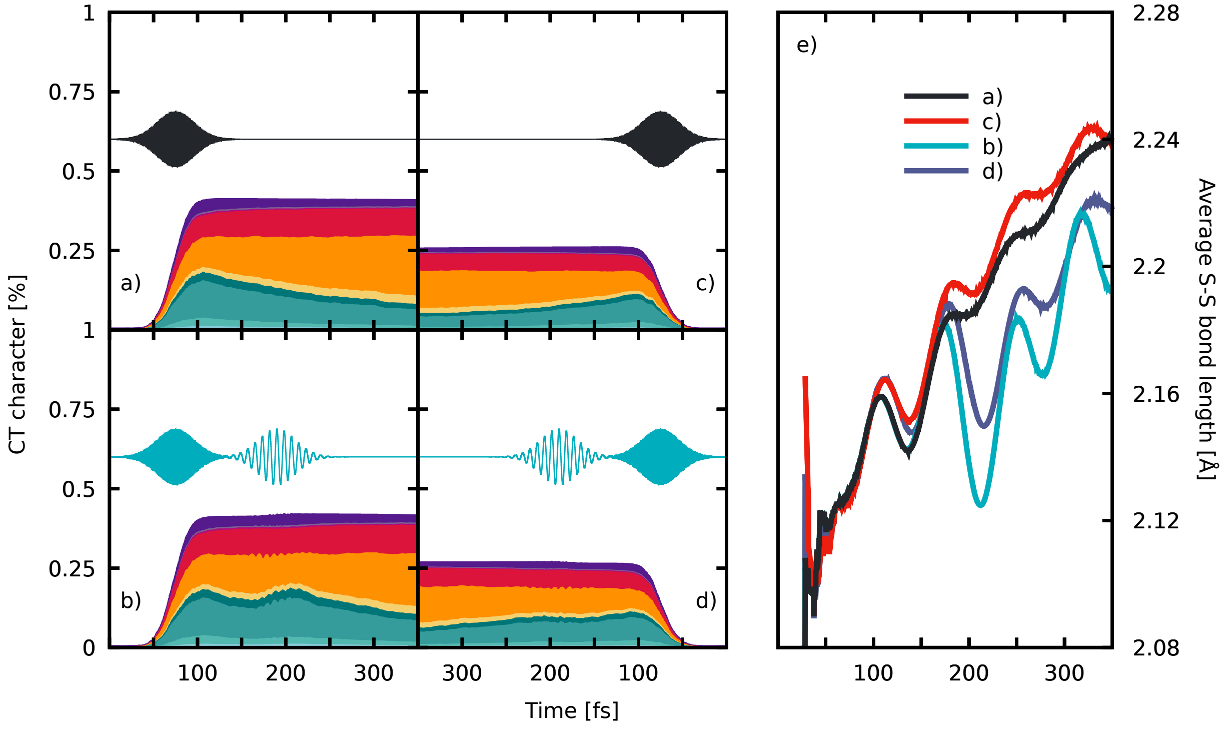

Figure S4: a) Time-resolved population after, (a) a pump$_{UV}$ pulse x-polarized, (b) a pump$_{UV}$-pump$_{IR}$ sequence x-polarized, (c) a pump$_{UV}$ pulse randomly polarized, and d) a pump$_{UV}$-pump$_{IR}$ sequence randomly polarized. e) Corresponding time-evolution of the S–S bond length. All laser pulses have a FWHM=50 fs.

S4a and c, for polarized along x or randomly) are very similar. In the S–S bond length for (Figure S4e), a slightly larger average is found for a randomly orientated electric field but overall the dynamics is very similar.

In the case of the pump$_{UV}$-pump$_{IR}$ sequence we also see a reduction in the amount of excited trajectories using randomized polarization vectors. However, the IR pulse results in a smaller increase of MLCT character than for a similar pulse along x. The average S–S bond length of excited trajectories is also less influenced by the control IR pulse but yields qualitatively similar results. Overall, we can conclude that the choice of the x direction for the electric field only enhance a stronger interaction with the ruthenium complex but dynamics is not affected. In an experiment, where the molecules are randomly oriented, only a smaller amount of those would be excited but the dynamics is expected to be qualitatively the same.

S6 Influence of adapting kinetic energy on a hop

In the main manuscript, the kinetic energy of a surface hopping trajectory was preserved during a hopping event. Therefore, the total energy in a trajectory is not conserved but instead increases for hops to higher lying surfaces and decreases for switches to lower energy surfaces. This methodology has been found[11,12,20] to give detrimental results for hops induced via non-adiabatic couplings as the presence of frustrated hops and the instantaneous adjustment of the kinetic energy is said to be important to mimic quantum behavior with the non-adiabatic coupling vector being the best choice for the direction of the velocity adjustment. However, when surface-hopping dynamics includes explicit laser fields, the overall energy conservation does not strictly apply, as hops caused by interaction with the electric field represent absorption or stimulated emission of a photon, associated with an allowed change in total energy. The presence of low-energy laser fields coupled with the propagation in a field-dependent basis invalidates using criteria to differentiate field- and non-adiabatic-induced. Hence, here the hops were not adjusted for energy.

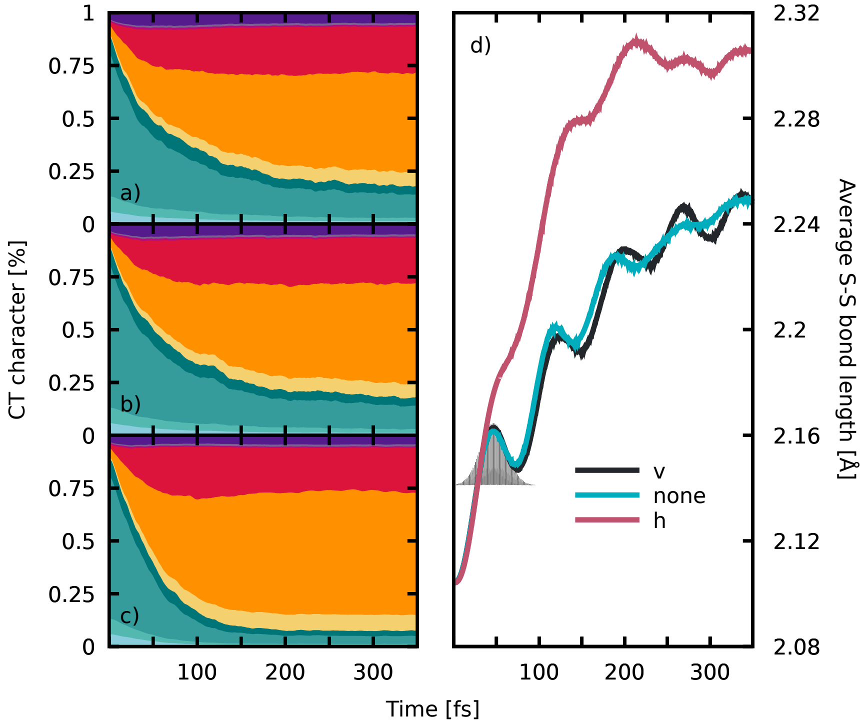

Figure S5: a-c) Time-resolved populations after trajectories are started directly in the electronic excited states. The difference between the sets is that the velocity vector (v) is rescaled at a hopping event to conserve the total energy in a) while b) does not show conservation of energy at a transition between surfaces (none) and c) uses the non-adiabatic coupling vector (h) to adjust the kinetic energy. d) Time evolution of the S–S bond length. The intensity of the pump pulse that was used for dynamics including electric fields is fitted as best as possible to the first maximum of the S–S bond length (see Section S3 for the corresponding discussion).

Still, we found interesting to investigate how large is the influence of adapting or not the

kinetic energy, for which two sets of trajectories have been simulated starting in the excited states, differing only in their treatment of velocity adjustment. Accordingly, we prepared two sets of 1000 trajectories, which for convenience, are stochastically selected based on the relative oscillator strength in the energy range of 2.85 to 3.05 eV and projected onto the corresponding excited states (i.e. no explicit laser pump pulse is used). One set of trajectories is rescaled along the velocity vector (v) at a hop, another set is not rescaled at all (none) and a final set uses the non-adiabatic coupling vectors (h) between the involved states to adjust the kinetic energy along. The resulting time-resolved populations are presented in Figure S5a-c. Interestingly, tiny deviations can be observed for the first two sets of dynamics. Only when using the non-adiabatic coupling vector to rescale, distinct and larger increase in MSCT states is observed, indicating that population of low-lying triplet states increases faster in this protocol. Also when looking at the S–S bond length (Figure S5d), deviations between both the first two protocols are negligible and occur only at later times. Not rescaling the velocities results in a stronger broadening of the oscillations of the S–S bond length compared to the adjustment along the full velocity vector. However, when using the non-adiabatic coupling vectors for energy conservation, a very steep increase in S–S bond length is observed, reaching a maximum value of around 2.31 Å after 200 fs from where on it stays almost constant. This value is very close to the equilibrium bond distance found at the T_1 minimum structure of the employed LVC model (2.34 Å), hinting that almost all trajectories are in the T_1 minimum after 200 fs. The differences between the dynamics and both the dynamics without energy adjustment and adjustment along the full velocity vector are twofold: when using the non-adiabatic coupling vectors, on the one side, the kinetic energy along specific bonds is increased when descending down the multitude of states, potentially increasing the kinetic energy along the S–S bond. On the other side, as the active state tries to switch to states with higher potential energy, frustrated hops are encountered when the kinetic energy along the non-adiabatic coupling vector is insufficient. This happened a total of 7231 times when using the non-adiabatic coupling vector and 0 times in the case of the full velocity vector which always contained enough energy (more than 7 eV in most cases) to accommodate the respective energy change. Therefore, on average 7 hops to higher-lying states have been frustrated per trajectory, barricading access to states that potentially feature larger MLCT character as they are generally located at higher energy and restricting the space of active states to lower energy states of predominantly MSCT character. We note that the non-adiabatic coupling vector has been commonly deemed the best and most physically sound choice for rescaling direction.[11,12,17,18]

In summary, we find that the differences between not rescaling or rescaling along the full velocity vector are very small. Overall, the energetic closeness of the excited states in this transition metal complex results in a large number of hops with small energy gaps. When distributing this small amount of energy using the full velocity vector, very small changes to the momenta of each single atom are observed. Therefore, rescaling along the full velocity vector conserves the total energy, but gives very similar results to not rescaling the kinetic energy at all. Adjusting the kinetic energy along the non-adiabatic coupling vector is found to change the speed upon which the S–S bond elongates as well the speed of the deactivation mechanics and would be the preferred treatment for hops that are not induced by an external field. Unfortunately, it is not possible to properly distinguish between field-induced hops and hops due to non-adiabatic couplings and spin-orbit couplings. Therefore, in order to not interfere with field-induced hops the simplest choice of not conserving the total energy was chosen for all encountered hops.

References

[1] Mai, S.; Richter, M.; Heindl, M.; Menger, M. F. S. J.; Atkins, A.; Ruckenbauer, M.; Plasser, F.; Ibele, L. M.; Kropf, S.; Oppel, M.; Marquetand, P.; González, L. SHARC2.1: Surface Hopping Including Arbitrary Couplings — Program Package for Non-Adiabatic Dynamics. sharc-md.org, 2019.

[2] Richter, M.; Marquetand, P.; González-Vázquez, J.; Sola, I.; González, L. SHARC: ab Initio Molecular Dynamics with Surface Hopping in the Adiabatic Representation Including Arbitrary Couplings. *J. Chem. Theory Comput.* **2011**, *7*, 1253–1258.

[3] Mai, S.; Marquetand, P.; González, L. A general method to describe intersystem crossing dynamics in trajectory surface hopping. *Int. J. Quantum Chem.* **2015**, *115*, 1215–1231.

[4] Fiedlschuster, T.; Handt, J.; Gross, E. K. U.; Schmidt, R. Surface Hopping in Laser-Driven Molecular Dynamics. *Phys. Rev. A* **2017**, *95*, 063424.

[5] Bajo, J. J.; González-Vázquez, J.; Sola, I. R.; Santamaria, J.; Richter, M.; Marquetand, P.; González, L. Mixed Quantum-Classical Dynamics in the Adiabatic Representation To Simulate Molecules Driven by Strong Laser Pulses. *J. Phys. Chem. A* **2012**, *116*, 2800–2807.

[6] Fiedlschuster, T.; Handt, J.; Schmidt, R. Floquet Surface Hopping: Laser-Driven Dissociation and Ionization Dynamics of H_2^+. *Phys. Rev. A* **2016**, *93*, 053409.

[7] Zhou, Z.; Chen, H.-T.; Nitzan, A.; Subotnik, J. E. Nonadiabatic Dynamics in a Laser Field: Using Floquet Fewest Switches Surface Hopping To Calculate Electronic Populations for Slow Nuclear Velocities. *J. Chem. Theory Comput.* **2020**, *16*, 821–834.

[8] Granucci, G.; Persico, M.; Toniolo, A. Direct Semiclassical Simulation of Photochemical Processes with Semiempirical Wave Functions. *J. Chem. Phys.* **2001**, *114*, 10608–10615.

[9] Granucci, G.; Persico, M. Critical Appraisal of the Fewest Switches Algorithm for Surface Hopping. *J. Chem. Phys.* **2007**, *126*, 134114.

[10] Granucci, G.; Persico, M.; Zoccante, A. Including Quantum Decoherence in Surface Hopping. *J. Chem. Phys.* **2010**, *133*, 134111.

[11] Tully, J. C. Molecular Dynamics with Electronic Transitions. *J. Chem. Phys.* **1990**, *93*, 1061–1071.

[12] Barbatti, M. Velocity Adjustment in Surface Hopping: Ethylene as a Case Study of the Maximum Error Caused by Direction Choice. *J. Chem. Theory Comp.* **2021**, *17*, 3010–3018.

[13] Bajo, J. J.; Granucci, G.; Persico, M. Interplay of Radiative and Nonradiative Transitions in Surface Hopping with Radiation-Molecule Interactions. *J. Chem. Phys.* **2014**, *140*, 044113.

[14] Topaler, M. S.; Allison, T. C.; Schwenke, D. W.; Truhlar, D. G. Test of Trajectory Surface Hopping Against Accurate Quantum Dynamics for an Electronically Nonadiabatic Chemical Reaction. *J. Phys. Chem. A* **1998**, *102*, 1666–1673.

[15] Hack, M. D.; Jasper, A. W.; Volobuev, Y. L.; Schwenke, D. W.; Truhlar, D. G. Quantum Mechanical and Quasiclassical Trajectory Surface Hopping Studies of the Electronically Nonadiabatic Predissociation of the Ã State of NaH_2. *J. Phys. Chem. A* **1999**, *103*, 6309–6326.

[16] Heindl, M.; González, L. Validating Fewest-Switches Surface Hopping in the Presence of Laser Fields. *J. Chem. Phys.* **2021**, *154*, 144102.

[17] Plasser, F.; Mai, S.; Fumanal, M.; Gindensperger, E.; Daniel, C.; González, L. Strong Influence of Decoherence Corrections and Momentum Rescaling in Surface Hopping Dynamics of Transition Metal Complexes. *J. Chem. Theory Comput.* **2019**, *15*, 5031–5045.

[18] Ibele, L. M.; Curchod, B. F. E. A Molecular Perspective on Tully Models for Nonadiabatic Dynamics. *Phys. Chem. Chem. Phys.* **2020**, *22*, 15183–15196.

[19] Trofimov, B. A.; Sinegovskaya, L. M.; Gusarova, N. K. Vibrations of the S–S bond in elemental sulfur and organic polysulfides: a structural guide. *J Sulfur Chem* **2009**, *30*, 518–554.

[20] Agostini, F.; Abedi, A.; Suzuki, Y.; Min, S. K.; Maitra, N. T.; Gross, E. K. U. The exact forces on classical nuclei in non-adiabatic charge transfer. *J. Chem. Phys.* **2015**, *142*, 084303.
